# Neurocan Inhibits Semaphorin 3F Induced Dendritic Spine Remodeling Through NrCAM in Cortical Neurons

**DOI:** 10.3389/fncel.2018.00346

**Published:** 2018-10-09

**Authors:** Vishwa Mohan, Elliott V. Wyatt, Ingo Gotthard, Kristen D. Phend, Simone Diestel, Bryce W. Duncan, Richard J. Weinberg, Ashutosh Tripathy, Patricia F. Maness

**Affiliations:** ^1^Department of Biochemistry and Biophysics, The University of North Carolina at Chapel Hill, Chapel Hill, NC, United States; ^2^Human Metabolomics, Institute of Nutrition and Food Sciences, University of Bonn, Bonn, Germany; ^3^Department of Cell Biology and Physiology, School of Medicine, The University of North Carolina at Chapel Hill, Chapel Hill, NC, United States

**Keywords:** neurocan, NrCAM, semaphorin, perineuronal net, dendritic spine pruning

## Abstract

Neurocan is a chondroitin sulfate proteoglycan present in perineuronal nets, which are associated with closure of the critical period of synaptic plasticity. During postnatal development of the neocortex dendritic spines on pyramidal neurons are initially overproduced; later they are pruned to achieve an appropriate balance of excitatory to inhibitory synapses. Little is understood about how spine pruning is terminated upon maturation. NrCAM (Neuron-glial related cell adhesion molecule) was found to mediate spine pruning as a subunit of the receptor complex for the repellent ligand Semaphorin 3F (Sema3F). As shown here in the postnatal mouse frontal and visual neocortex, Neurocan was localized at both light and electron microscopic level to the cell surface of cortical pyramidal neurons and was adjacent to neuronal processes and dendritic spines. Sema3F-induced spine elimination was inhibited by Neurocan in cortical neuron cultures. Neurocan also blocked Sema3F-induced morphological retraction in COS-7 cells, which was mediated through NrCAM and other subunits of the Sema3F holoreceptor, Neuropilin-2, and PlexinA3. Cell binding and ELISA assays demonstrated an association of Neurocan with NrCAM. Glycosaminoglycan chain interactions of Neurocan were required for inhibition of Sema3F-induced spine elimination, but the C-terminal sushi domain was dispensable. These results describe a novel mechanism wherein Neurocan inhibits NrCAM/Sema3F-induced spine elimination.

## Introduction

Dendritic spines of cortical pyramidal neurons are the principal site of excitatory synapse formation. Spines undergo dynamic changes during development, including rapid spinogenesis in early postnatal life, followed by substantial pruning during adolescence (Huttenlocher, 1979; McAllister, 2007; Petanjek et al., 2011; Bian et al., 2015). Spines are stabilized in the juvenile-to-adult transition but remain dynamic (though with slower turnover rates) in the adult. Recently, we showed that NrCAM, a neural cell adhesion molecule of the L1 family, regulates Semaphorin 3F (Sema3F)-mediated dendritic spine pruning in the mouse prefrontal and visual cortex during adolescence (Demyanenko et al., 2014; Mohan et al., 2018). NrCAM binds directly to Neuropilin-2 (Npn2), which associates with Plexin A3 (PlexA3) to form a Sema3F holoreceptor complex that mediates spine elimination (Tran et al., 2009; Mohan et al., 2018). In the presence of Sema3F, NrCAM promotes higher order clustering of Npn2 and PlexA3 on the neuronal membrane essential for spine pruning (Mohan et al., 2018).

Mechanisms governing the active phase of spine remodeling in adolescence and transition to more stable spines in adult brain are not well-defined. Perineuronal nets (PNNs) are mesh-like structures that surround neurons and arise postnatally around the time when synaptic contacts are stabilized (Miyata and Kitagawa, 2017). PNNs are composed of chondroitin sulfate proteoglycans (CSPGs) with glycosaminoglycan (GAG) side chains, hyaluronic acid, tenascin-R, and link proteins that coalesce to form molecular aggregates around neuronal cell bodies and processes. PNNs have been shown to restrict neural plasticity in brain regions including the visual cortex (Pizzorusso et al., 2002; Hensch, 2005; Pyka et al., 2011; de Vivo et al., 2013) and amygdala (Gogolla et al., 2009; Hylin et al., 2013). This restriction is partly reversible, as enzymatic degradation of GAG chains of CSPGs by chondroitinase can restore aspects of juvenile plasticity to adult circuits (Miyata and Kitagawa, 2017). Of particular interest, spine dynamics in immature cortical pyramidal neurons diminish with maturation but can be restored by degradation of PNNs (de Vivo et al., 2013).

Neurocan is a CSPG that is a prominent organizer of PNNs in the neocortex. Genetic studies in humans have identified Neurocan as a potential risk factor for schizophrenia, bipolar disorder (Muhleisen et al., 2012; Schultz et al., 2014; Wang et al., 2016), and dyslexia (Einarsdottir et al., 2017). Our interest in mechanisms regulating spine density in the developing cortex led us to investigate molecules that may mediate the reduction of spine remodeling that occurs with maturation. We hypothesized that Neurocan may inhibit spine remodeling by interfering with the functional interaction between Sema3F and NrCAM. Our results show that Neurocan inhibits Sema3F-induced spine remodeling in cortical neuronal cultures, and localizes to neuronal plasma membranes and extracellular space during maturation of the mouse neocortex. These data suggest a novel role for Neurocan as a developmental brake for spine remodeling mediated by Sema3F and NrCAM in the maturing mouse neocortex.

## Materials and Methods

### Mice

Wild type (WT) and NrCAM null mutant mice in the C57BL/6 genetic background were maintained in a 12-h day and night cycle environment with *ad libitum* availability of chow diet and water. For labeling postmitotic pyramidal neurons in the cerebral cortex, Nex1-CreERT2 mice were crossed with the Ai9 reporter strain (both in C57Bl/6) to generate a tamoxifen-inducible reporter line of mice expressing tdTomato in postmitotic pyramidal neurons under the control of Nex1-Cre as previously characterized (Agarwal et al., 2012; Mohan et al., 2018). Recombination-induced expression of tdTomato in postmitotic pyramidal neurons was achieved by daily injections of tamoxifen from postnatal day P10-13, as described (Agarwal et al., 2012; Mohan et al., 2018). All animal experiments were approved by the Institutional Animal Care and Use Committee of The University of North Carolina School of Medicine at Chapel Hill (IACUC Protocol # 15-114). Mice were handled according to the University of North Carolina Institutional Animal Care and Use Committee policies and in accordance with NIH guidelines for humane care and use of laboratory animals.

### Immunoblotting

Lysates of mouse cortex (P7, P14, P21, and P80) and cell cultures were prepared in lysis buffer (1% Brij98, 10 mM Tris-Cl pH 7.0, 150 mM NaCl, 1 mM NaEDTA, 1 mM NaEGTA, 200 μM Na3VO4, 10 mM NaF, and 1X protease inhibitors (Sigma-Aldrich). Lysates (50 μg) were subjected to Western blotting with the following antibodies: anti-NrCAM (1:1000, Abcam), anti-Neurocan (1:500, R&D), anti-Sema3F (1:500, Millipore), and anti-β actin (1:1000, Millipore). Blots were developed with HRP-tagged secondary antibodies (1:5000, Jackson Immunoresearch) using Western Bright ECL Substrate (Advansta) and bands quantified by densitometry.

### Immunostaining

For immunostaining, neuronal cultures transfected with pCAGGS-IRES-mEGFP were fixed at DIV14 in 4% paraformaldehyde (PFA), permeabilized with Triton X-100, blocked in 10% horse or donkey serum, and labeled with chicken anti-GFP (Abcam). Secondary anti-chicken Alexa Fluor 488 antibodies (1:500) were added for 1 h before mounting and confocal imaging. For Neurocan localization, 100 μm coronal brain sections were prepared on vibratome from Nex1-CreERT2:Ai9 mice (P18 and P80) expressing tdTomato in pyramidal neurons. Serial 100 μm vibratome sections from P18 and P80 brain were matched for level based on rostrocaudal distance from the anterior end of the brain. Samples were blocked in PBS, 10% donkey serum, 0.3% Triton X100, then incubated with Neurocan antibodies (1:500, R&D) for 24 h at 4°C, then with anti-sheep Alexa Fluor 488 secondary antibody (1:500). After washing, sections were mounted with Prolong Gold anti-fade reagent (Invitrogen) and imaged using a Zeiss LSM 700 confocal microscope. All images were captured using identical microscope settings, we kept the total z thickness (7.35 μm) as well as thickness of single optical sections (0.35 μm) same for all samples. tdTomato (red) fluorescence was excluded from analysis. The intensity of total Neurocan fluorescence observed in the green channel was quantified for each image after auto-thresholding without regard to tdTomato fluorescence. Quantification of pixel intensity was performed blindly using ImageJ software (NIH).

### Neurocan Immunogold Labeling and Electron Microscopy

C57BL/6 WT mice (P18 and P80) were anesthetized and perfused transcardially with phosphate buffer (0.15 M sodium phosphate, pH 7.4) and postfixed in 4% PFA, 0.1% glutaraldehyde in PBS. Coronal vibratome sections (50 μm) were subjected to pre-embedding immunogold labeling with silver enhancement using Neurocan antibodies and streptavidin-nanogold (Nanoprobes 2016), as we previously described (Sullivan et al., 2018). Sections were silver-enhanced using HQ silver enhancement kit (Nanoprobes 2012), and postfixed in osmium tetroxide. Sections were then stained with uranyl acetate and infiltrated with resin. Tissue was sectioned (50–60 nm), collected on 300 mesh nickel or copper grids, counterstained with uranyl acetate and Sato’s lead and examined with a Tecnai 12 transmission electron microscope.

### Spine Retraction Assay in Cortical Neuronal Cultures

Cortical neuronal cultures were prepared from mouse embryos (E15.5) and plated on laminin, poly-D-lysine coated chamber slides. Neurons were maintained in neurobasal medium (Gibco) supplemented with B27 and antibiotics. Cells were transfected with plasmid pCAGGS-IRES-mEGFP at DIV11 using Lipofectamine 2000 (Thermo Fisher Scientific). As described in (Mohan et al., 2018), transfected cells at DIV14 were treated with 3 nM Sema3F-Fc (R&D) or Fc (Abcam) for 30 min. Where indicated, cultures were pre-treated for 30 min with 8–20 nM full length recombinant human Neurocan (Glu23-Cys1321, R&D) or a mouse Neurocan fragment (Asp23-Asp637, R&D), which lacks the C-terminal sushi domain and approximately half of the GAG-modified region. Cells were fixed in 4% PFA, permeabilized with Triton X-100, blocked in 10% serum, and labeled with anti-GFP to enhance visualization of spines. Spine densities were scored on the first branch of multiple apical dendrites using Neurolucida software. Spine analysis was blinded. Data was collected from confocal images of EGFP-labeled neurons with pyramidal morphology in each of four replicate cultures. Mean spine densities ± SEM were compared by the *t*-test (2-tailed, unequal variances, *p* < 0.05). To test the effect of GAG chain digestion, Neurocan was incubated in solution with 0.1 units/μg chondroitinase ABC (chABC, Sigma) at 37°C for 1.5 h, followed by heat inactivation of enzyme at 100°C for 10 min. Efficacy of GAG digestion was assessed by immunoblotting.

### COS-7 Cell Retraction Assay

COS-7 cells were plated on eight well chamber slides (25,000 cells/well) coated with poly-D-lysine. The following day cells were transfected with plasmids expressing NrCAM (pCMV6), Npn2 (pCOS), and/or PlexA3-EGFP (pCAGGS-PlexA3-IRES-mEGFP). At 48 h after transfection cells were treated with 3 nM Sema3F-Fc or Fc for 30 min, subjected to immunofluorescence staining for GFP and imaged on confocal microscope. The particle tool of ImageJ was used after auto-thresholding to measure the area of individual labeled cells. The software auto-detects the boundary of cells in thresholded images. Cells between 500 and 1000 μm^2^ were classified as retracted (collapsed), and those greater than 1000 μm^2^ were classified as non-retracted. The mean percent of collapsed cells relative to the total GFP-positive cells was calculated from three experiments, 10 images per condition, and compared by the *t*-test for significant differences (^∗^*p* < 0.05, *n* = 3 from ∼200 cells/image). Preliminary dose response experiments showed that Neurocan (4–40 nM) effectively inhibited the Sema3F-Fc-induced COS-7 cell retraction response; we selected 8 nM for subsequent studies.

### Cell Binding and ELISA Assays

COS-7 cells were transfected with NrCAM in pCMV6 or vector alone, together with pCAGGS-IRES-mEGFP. Cortical neurons from NrCAM homozygous null mice were transfected on DIV11 with pCAGGS-NrCAM-IRES-mEGFP or vector alone. At 48 h post-transfection, cells were treated with 20 nM recombinant Neurocan at 37°C for 30 min. To assay Neurocan binding to the cell surface, cultures were fixed and subjected to indirect immunofluorescence staining with antibodies to Neurocan and secondary Alexa Fluor-conjugated antibodies. Single optical section images were taken on the confocal microscope using the same settings for each condition, and the fluorescence intensity of staining measured using Image J.

ELISA was performed to measure the binding between NrCAM-Fc (R&D) and alkaline-phosphatase-tagged Neurocan (Neurocan-AP). The Neurocan-AP fusion protein construct was generated by PCR amplification of full-length mouse Neurocan cDNA (Accession: BC065118), followed by subcloning into the APtag5 vector so that the alkaline phosphatase (AP) tag was inserted at the N-terminus of Neurocan (Shen et al., 2009). Neurocan-AP or AP control proteins were harvested from conditioned media of HEK293T cells transfected with Neurocan-APtag5 or APtag5 plasmids after 72 h. Media was clarified by centrifugation and filtered through a 0.45 μm filter. Detailed methods of APtag5 protein production have been described (Flanagan and Cheng, 2000). The ELISA was carried out essentially as described for Neurocan-AP binding to protein tyrosine phosphatase sigma (Shen et al., 2009). Protein A plates (Pierce) were coated with 1 μM NrCAM-Fc or Fc in HBSS-20 mM HEPES, pH 7.0 for 2 h at room temperature. Blocking was performed with SuperBlock (Thermo Fisher Scientific). After washing in HBSS-20 mM HEPES, wells were incubated with 10 μM Neurocan-AP or AP for 1.5 h at RT. Reaction product was developed using p-nitrophenylphosphate to detect AP activity. Optical density measurements were taken at 405 nm on an ELISA plate reader.

To assess direct binding of Neurocan with semaphorins, 1 μg of full length recombinant Neurocan (R&D) was incubated with 1 μg of either control Fc, Sema3A-Fc, or Sema3F-Fc in Tris-buffered saline for 1 h at 37°C. Protein A/G Sepharose beads were used to pull down Fc proteins for detection of bound Neurocan by immunoblotting with Neurocan antibodies. Blots were reprobed with anti-Fc antibodies.

## Results

### Expression and Localization of Neurocan in Frontal and Visual Cortex

**FIGURE 1:**
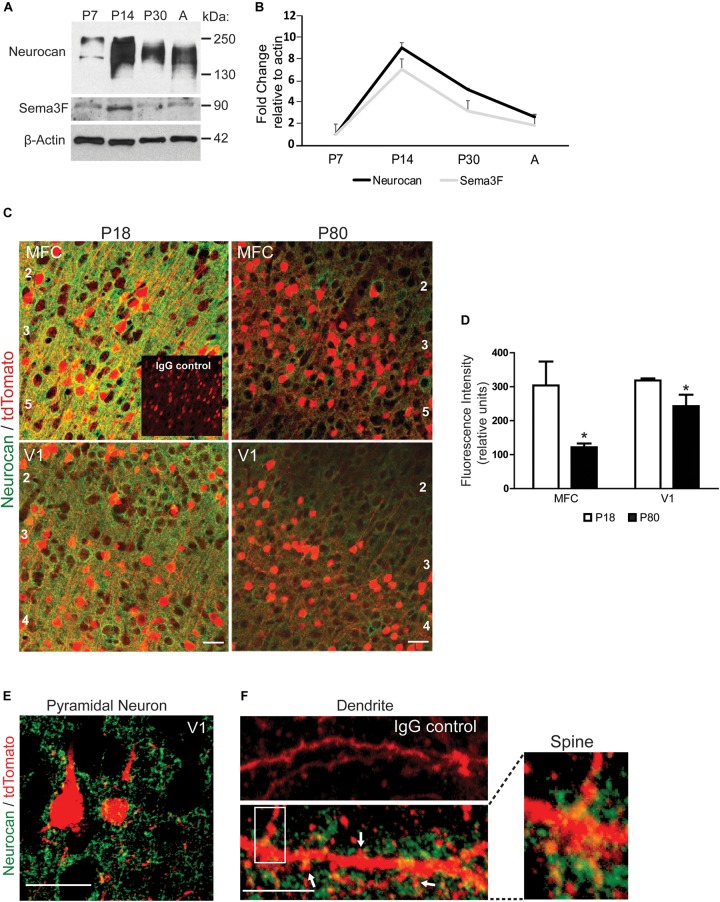
Expression of Neurocan in developing mouse neocortex. **(A)** Representative immunoblots of Neurocan and Sema3F in lysates of cerebral cortex (50 μg) from postnatal (P7, P14, and P30) and adult brain (A). β-Actin served as loading control. **(B)** Relative levels of Neurocan and Sema3F in cerebral cortex at each developmental stage (*n* = 3) normalized to β-actin. Mean ± SEM are shown. **(C)** Immunofluorescence staining for Neurocan (green) and tdTomato (red) in coronal sections from MFC and V1, in Nex1-CreERT2:Ai9 mice at adolescent (P18) and adult (P80) stages. Nonimmune IgG control is shown as an inset. Cortical layers are numbered. Scale bar = 50 μm. **(D)** Fluorescence intensity of Neurocan immunostaining in MFC and V1 at P18 and P80 (*n* = 10 images for each condition from three brains). Mean differences (±SEM) in Fc versus Sema3F-Fc treated cultures were compared for significance (^∗^*t*-test and *p* < 0.05). **(E)** Representative image showing Neurocan immunostaining (green) surrounding tdTomato-positive pyramidal neurons in V1, of Nex1-CreERT2:Ai9 mice. Scale bar = 50 μm. **(F)** Upper panel, normal IgG; lower panel, Neurocan immunostaining (green) around dendrites with spines (scale bar = 10 μm). Far right panel shows magnified view of boxed area.

To evaluate the postnatal expression of Neurocan in developing mouse brain, cortical lysates were analyzed at P7, P14, P30, and adult by Western blotting. Neurocan was detected as a broad band from 150 to 250 kDa reflecting GAG-modification (**Figure 1A**). Quantification showed that Neurocan expression in the cortex increased markedly (∼8 fold) from P7 to P14, declined at P30, and persisted in adulthood (**Figures 1A,B**). The level of Sema3F (90 kDa) in the developing cortex paralleled that of Neurocan with a ∼6 fold increase from P7 to P14 then a decrease with maturation (**Figures 1A,B**). To determine if Neurocan was localized to cortical pyramidal neurons at adolescent (P18) and adult (P80) stages, immunofluorescence staining for Neurocan was performed in the medial frontal cortex (MFC) and primary visual cortex (V1). These cortical regions have been shown to be sites of spine density regulation on apical dendrites of pyramidal neurons by Sema3F and NrCAM/Npn2/PlexA3 (Mohan et al., 2018). To identify pyramidal neurons, we used a tamoxifen-inducible reporter line of mice expressing tdTomato specifically in postmitotic pyramidal neurons under the control of Nex1-CreERT2 promoter (Agarwal et al., 2012; Mohan et al., 2018). At both P18 and P80, Neurocan labeling in the MFC and V1 was prominent around cell bodies and processes of tdTomato-positive pyramidal neurons throughout the cortical layers, in addition to other cells (**Figure 1C**). Nonimmune IgG (IgG) staining was negligible. The intensity of Neurocan immunofluorescence (green channel only, excluding tdTomato) was quantified in coronal sections matched for level from confocal images obtained under identical settings. Neurocan immunofluorescence levels throughout the cortical layers were significantly greater at P18 than P80 in both the MFC and V1 (**Figure 1D**). At higher magnification Neurocan labeling of pyramidal neurons at P18 in the MFC appeared to be diffusely localized around soma, dendrites, and extracellular space (**Figure 1E**). Neurocan was also evident as a diffuse net at or near the membrane of dendritic branches, including in the vicinity of dendritic spines (arrows, **Figure 1F**).

**FIGURE 2:**
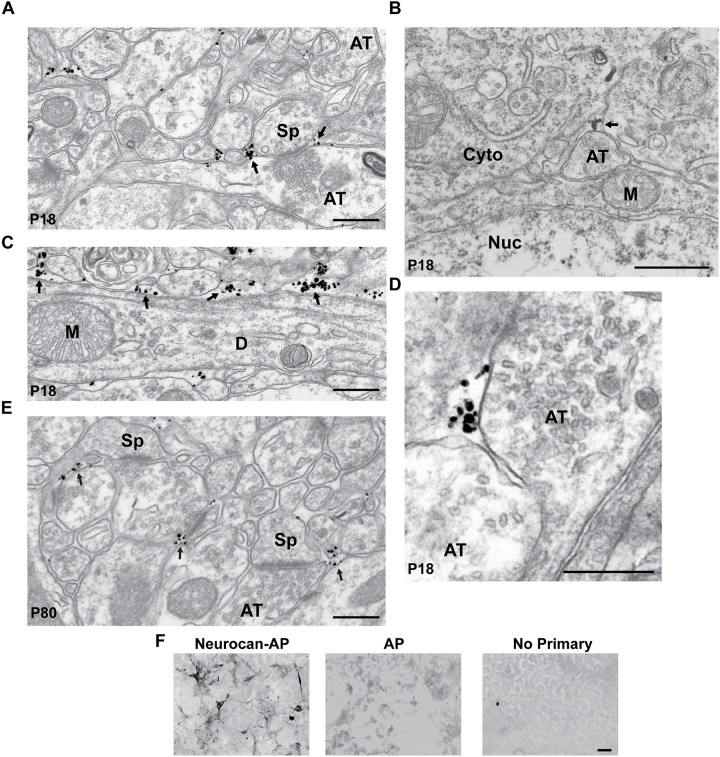
Localization of Neurocan in mouse medial frontal cortex (MFC) by immunogold labeling and electron microscopy. **(A)** Electron micrograph of MFC layer 2/3 at P18, showing immunogold labeling of Neurocan near the plasma membrane adjacent to a spine (Sp) and axon terminal (AT; arrows). **(B)** Neurocan labeling in the extracellular space near an axon terminal (AT; arrow) at P18 [Nucleus (Nuc) and cytoplasm (Cyto)]. **(C)** Accumulation of Neurocan (arrows) in extracellular space and along the plasma membrane of a dendrite (D) at P18. Mitochondria (M) were unlabeled. **(D)** Neurocan labeling adjacent to axon terminals (AT) at P18. **(E)** Neurocan labeling at neck of spine (Sp) and near excitatory synapses (arrows) at P80. Scale bar = 1 μm. **(F)** Validation of Neurocan antibody specificity by immunoperoxidase staining of COS-7 cells transfected with Neurocan-AP or AP alone in the APtag5 vector, using Neurocan antibodies or no primary antibody. An antibody dilution series was carried out in pilot experiments. Scale bar = 50 μm.

The subcellular localization of Neurocan was investigated in greater detail in the MFC by immunogold labeling at the electron microscope level. At P18, labeled Neurocan was observed near the neuronal membrane adjacent to excitatory, asymmetric synapses of dendritic spines (Sp; **Figure 2A**, arrows) but it was not found directly within synaptic junctions. Neurocan was also evident at the membrane or extracellular space, and could be seen near presynaptic axon terminals (AT), which harbored synaptic vesicles (**Figures 2A,B,D**, arrows). Neurocan labeling was frequently clustered at or near dendritic membranes as well as the extracellular space (**Figure 2C**, arrows). At P80, Neurocan was observed at the plasma membrane near spines, and sometimes adjacent to asymmetric, excitatory synapses (**Figure 2E**). At both stages labeling was rarely present within the cytoplasm. The appropriate dilution of Neurocan antibodies and specificity was pre-assessed by immunoperoxidase staining of COS-7 cells transfected with plasmids expressing Neurocan-AP or control AP protein. Cells expressing Neurocan-AP were clearly stained with Neurocan antibodies, whereas cells expressing AP elicited minimal staining, as did omission of primary antibody (**Figure 2F**). In summary, Neurocan was expressed prominently in the postnatal MFC and V1, declining with maturation, and was localized near the plasma membrane of pyramidal cell processes and adjacent to dendritic spines.

### Neurocan Inhibits Sema3F-Induced Spine and Cell Retraction

**FIGURE 3:**
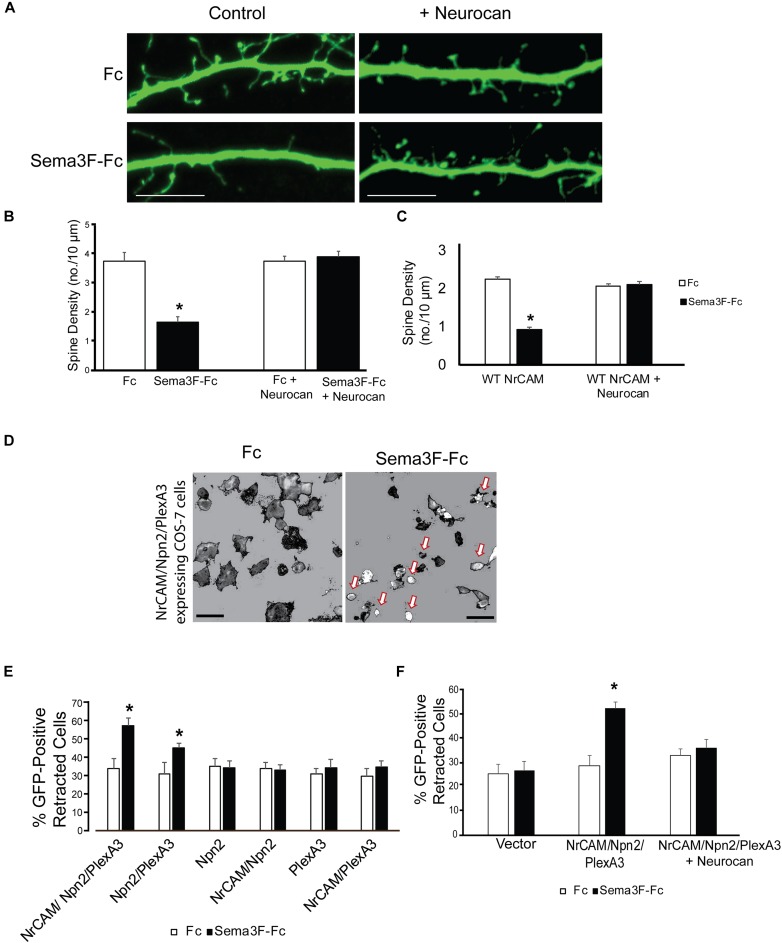
Neurocan inhibits Sema3F-induced spine and cell retraction. **(A)** Apical dendrites and spines of mouse cortical neurons expressing EGFP in cultures treated with Fc or Sema3F-Fc for 30 min. Pretreatment with 20 nM Neurocan for 30 min prevented Sema3F-Fc induced spine collapse but did not affect spine density in Fc-treated control cultures. Scale bar = 10 μm. **(B)** Quantification of experiment in panel **A**, showing significant reduction in mean spine density upon Sema3F-Fc treatment by Neurocan (*n* = 3, 10 neurons per condition; ^∗^*p* < 0.05, *t*-test). **(C)** Treatment with 8 nM Neurocan for 30 min also prevented Sema3F-Fc induced spine collapse and did not affect spine density in Fc-treated control cultures (*n* = 3, 10 neurons per condition; ^∗^*p* < 0.05, *t*-test). **(D)** Representative thresholded images showing morphological retraction of COS-7 cells expressing NrCAM, Npn2, PlexA3, and EGFP after Sema3F-Fc treatment compared to Fc. Arrows point to retracted cells. Scale bar = 100 μm. **(E)** Quantification of morphological cell retraction after Fc or Sema3F-Fc treatment of COS-7 cells expressing NrCAM, Npn2, and/or PlexA3 and EGFP in panel **D**. Results show percent retracted cells (*n* = 3 assays, ^∗^*p* < 0.05, and *t*-test). **(F)** Quantification of morphological retraction upon Fc or Sema3F-Fc treatment of COS-7 cells expressing NrCAM, Npn2, and PlexA3 with and without pre-treatment with 20 nM Neurocan for 30 min (*n* = 3 assays, ^∗^*p* < 0.05, and *t*-test).

To assess the potential of Neurocan to terminate spine remodeling during postnatal maturation, we tested the ability of Neurocan to inhibit Sema3F-induced spine retraction in cortical neurons in culture. In this assay (Peng and Tran, 2017; Mohan et al., 2018), dissociated cortical neurons from mouse embryos at E15.5 were transfected with pCAGGS-IRES-mEGFP at DIV11 and cultured to DIV14. Cells were pre-treated with or without Neurocan for 30 min, then stimulated with 3 nM Sema3F-Fc or Fc protein for 30 min. We used a concentration of Neurocan (20 nM) that exceeded the Kd for Neurocan binding to L1-CAM (∼1 nM) (Friedlander et al., 1994; Milev et al., 1998), effectively inhibited ephrinA5-induced axon terminal retraction in cortical neuron cultures (Sullivan et al., 2018), and was within the estimated physiological range in rodent brain, which varies with age and region (Rauch et al., 1992; Bhattacharyya et al., 2015). Analysis of spine density on apical dendrites of EGFP-labeled neurons showed that Neurocan (20 nM) blocked Sema3F-induced spine retraction, but had no effect on spine density of Fc-treated neurons (**Figures 3A,B**). Similar results in neuronal cultures with a lower spine density were obtained using a lower concentration of Neurocan (8 nM) (**Figure 3C**).

To evaluate the molecules involved, we used a heterologous assay in which morphological retraction of COS-7 cells can be induced by Sema3A-Fc or Sema4D-Fc (Turner and Hall, 2006). COS-7 cells were transfected with one or more plasmids expressing NrCAM, Npn2, PlexA3, then treated with 3 nM Sema3F-Fc or Fc for 30 min. COS-7 cells expressing all three Sema3F holoreceptor subunits NrCAM, Npn2, and PlexA3, showed the highest percent of retracted cells after treatment with Sema3F-Fc compared to Fc (**Figures 3D,E**). Cells expressing only Npn2 and PlexA3 also retracted upon Sema3F-Fc treatment but required NrCAM for maximal response (**Figure 3E**). Significant retraction was not observed in COS-7 cells expressing Npn2 or PlexA3 alone, or in cells doubly expressing NrCAM/Npn2 or NrCAM/PlexA3 (**Figure 3E**). Cells expressing only EGFP also did not respond to Sema3F-Fc (**Figure 3F**). A basal level of retracted cells was observed in each condition (25–30%), which may be due to changes in shape as a result of mitosis, migration, or cell heterogeneity. To determine if Neurocan negatively regulated Sema3F-induced cell retraction, COS-7 cells expressing NrCAM/Npn2/PlexA3 were pre-treated with 8 nM Neurocan, then stimulated with 3 nM Sema3F-Fc or Fc. Neurocan effectively blocked NrCAM/Npn2/PlexA3-dependent retraction to Sema3F-Fc (**Figure 3F**). These results demonstrated that Neurocan negatively regulates Sema3F-induced retraction through a mechanism involving the NrCAM/Npn2/PlexA3 receptor complex.

### NrCAM-Dependent Binding of Neurocan

**FIGURE 4:**
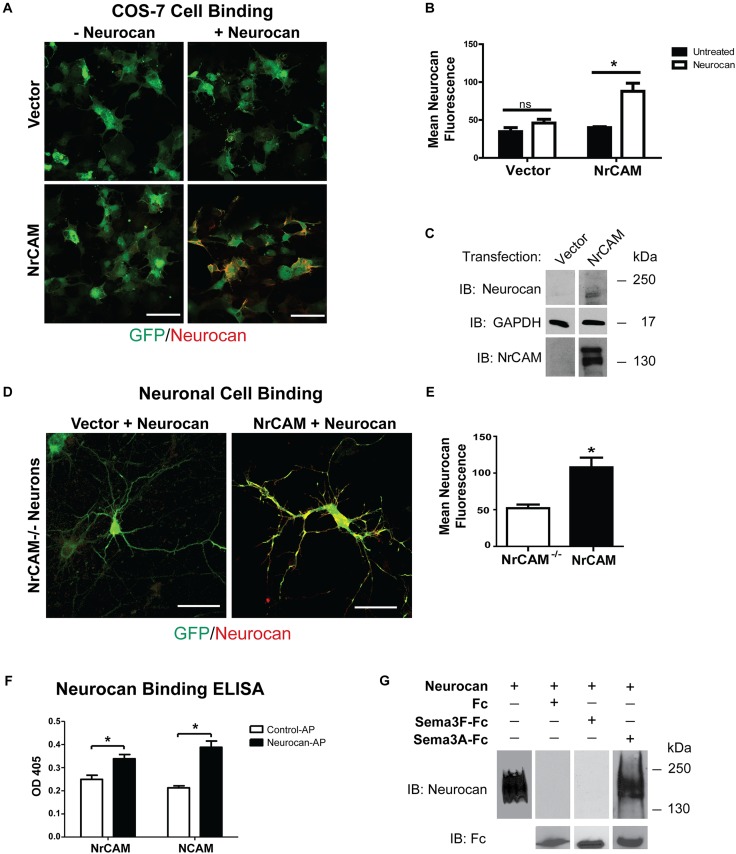
Cell binding and Neurocan interaction with NrCAM. **(A)** COS-7 cells transfected with vector alone (pCAGGS-IRES-mEGFP) or pCAGGS-NrCAM-IRES-mEGFP were pre-treated with 8 nM Neurocan, then fixed and subjected to immunofluorescence staining without permeabilization to detect surface-bound Neurocan (red). Scale bar = 100 μm. **(B)** Mean fluorescence intensity (±SEM) of Neurocan immunofluorescence staining on the surface of COS-7 cells, as shown in panel **A**. NrCAM-expressing cells treated with Neurocan showed significantly greater levels of bound Neurocan than untreated cells. Fluorescence intensity in cells with vector alone treated with Fc or Sema3F-Fc was non-significant (ns). ^∗^*p* > 0.05, *t*-test, *n* = 5 images each condition. **(C)** Lysates (50 μg) of cells transfected with vector alone or pCAGGS-NrCAM-IRES-mEGFP were treated with Neurocan as in panel **A**, and immunoblotted (IB) with Neurocan antibodies. Blots were reprobed with antibodies directed against GAPDH (loading control) or NrCAM (expression control). Representative immunoblots of three experiments are shown. **(D)** Mouse cortical neuron cultures from NrCAM null mice were transfected with vector alone or pCAGGS-NrCAM-IRES-EGFP, and pre-treated with 20 nM Neurocan before fixation and immunostaining to detect surface-bound Neurocan. In merged images of EGFP (green) and Neurocan (red), more Neurocan immunofluorescence was observed on the surface of neurons expressing NrCAM than on NrCAM null neurons with vector alone. Scale bar = 50 μm. **(E)** Mean fluorescence intensity (±SEM) of surface-bound Neurocan immunostaining on neurons in panel **D** NrCAM-expressing cells treated with Neurocan showed significantly greater levels of bound Neurocan than NrCAM-minus neurons. ^∗^*p* > 0.05, *t*-test, and *n* = 10 neurons per condition. **(F)** ELISA of Neurocan-AP or control AP protein binding to NrCAM-Fc or positive control NCAM-Fc on protein A-coated microtiter wells. AP binding was detected colorimetrically with p-nitrophenylphosphate. The mean (±SEM) optical densities (OD 405) of Neurocan-AP bound to NrCAM-Fc or NCAM-Fc were significantly greater than control AP (*t*-test and ^∗^*p* > 0.05). **(G)** Recombinant human Neurocan was incubated in Tris buffered saline with purified Fc, Sema3F-Fc, or Sema3A-Fc proteins, then complexes were pulled down with Protein A/G Sepharose beads. Immunoblotting for Neurocan showed no binding of Neurocan to Fc or Sema3F-Fc, whereas Neurocan bound effectively to Sema3A-Fc. Blots were reprobed with anti-Fc antibodies to demonstrate that equivalent amounts of Fc fusion proteins were pulled down. Recombinant Neurocan (left lane) ran as a broad band between 250 and 130 kDa.

We hypothesized that Neurocan negatively regulates Sema3F-mediated retraction by binding to the extracellular region of NrCAM at the cell surface. To test this, we examined the ability of Neurocan to bind to COS-7 cells transfected with NrCAM or vector alone, together with EGFP. COS-7 cells were treated 48 h post-transfection with or without 20 nM Neurocan for 30 min. Cells were washed extensively, fixed without permeabilization, and subjected to immunofluorescence staining with Neurocan antibodies. The amount of surface-bound Neurocan on EGFP-labeled cells was quantified by measuring the fluorescence intensity of Neurocan staining in single optical sections. Results showed that Neurocan bound at greater levels to the surface of cells that expressed NrCAM compared to cells without NrCAM transfected with vector alone (**Figures 4A,B**). The low level of fluorescence seen in untreated cells was likely nonspecific. Immunoblotting for Neurocan and GAPDH (loading control) in lysates of similarly treated cells, showed that Neurocan was detected in cells transfected with the NrCAM plasmid but not in control cells without NrCAM (**Figure 4C**).

To determine if Neurocan bound to neuronal cells expressing NrCAM, cortical neuron cultures from homozygous NrCAM null mutant embryos (E15.5) were transfected at DIV11 with pCAGGS-IRES-mEGFP or pCAGGS-NrCAM-IRES-mEGFP, and treated at DIV14 with 20 nM Neurocan for 30 min. After washing and fixation without permeabilization, neurons were immunostained to detect Neurocan on the cell surface. EGFP-labeled neurons expressing NrCAM exhibited a greater amount of surface-bound Neurocan than NrCAM-minus neurons, and quantitation showed that this difference was significant (**Figures 4D,E**). The lower level of Neurocan fluorescence on NrCAM-minus neurons may be due to interaction with other binding proteins on the cell surface.

Results of the cell binding assays showed that expression of NrCAM leads to increased binding of Neurocan to the cell surface. To assess a direct interaction of Neurocan with the NrCAM extracellular region, we developed an ELISA using purified, full-length mouse Neurocan fused to AP expressed in HEK293T cells. NrCAM-Fc protein was adsorbed to ELISA plates that were pre-coated with Protein A, then incubated with Neurocan-AP or AP control protein for 1.5 h. Binding was quantified by colorimetric detection of bound AP using p-nitrophenylphosphate. As a positive control, Neurocan-AP was also assayed for binding to NCAM, a different cell adhesion molecule known to engage Neurocan at its extracellular Ig2 domain (Sullivan et al., 2018). Neurocan-AP bound to NrCAM-Fc to a significantly greater extent than control AP (**Figure 4F**). Similar levels of Neurocan-AP binding to NCAM-Fc were observed. Taken together, these results support the conclusion that Neurocan binds to the extracellular region of NrCAM and inhibits Sema3F- mediated spine elimination in maturing pyramidal neurons of the mouse neocortex.

Sema3A is a class 3 semaphorin that has been reported to bind to chondroitin sulfates in PNNs but has not been shown to bind Neurocan (Carulli et al., 2013; Dick et al., 2013). The possibility of sequestration of Sema3F-Fc by Neurocan was tested by incubating Sema3F-Fc, Sema3A-Fc, or Fc proteins in Tris buffered saline (TBS) with purified recombinant mouse Neurocan *in vitro*. Fc-containing protein complexes were pulled down with Protein A/G-Sepharose and subjected to Western blotting to detect Neurocan present in the Protein A/G pull-downs. Results showed that Neurocan bound to Sema3A-Fc, but there was no detectable association with Sema3F-Fc or Fc alone (**Figure 4G**). Reprobing blots with anti-Fc antibodies confirmed that pull-downs contained approximately equivalent amounts of Fc-containing protein.

### Importance of Neurocan GAG Chains in Regulation of Spine Retraction

**FIGURE 5:**
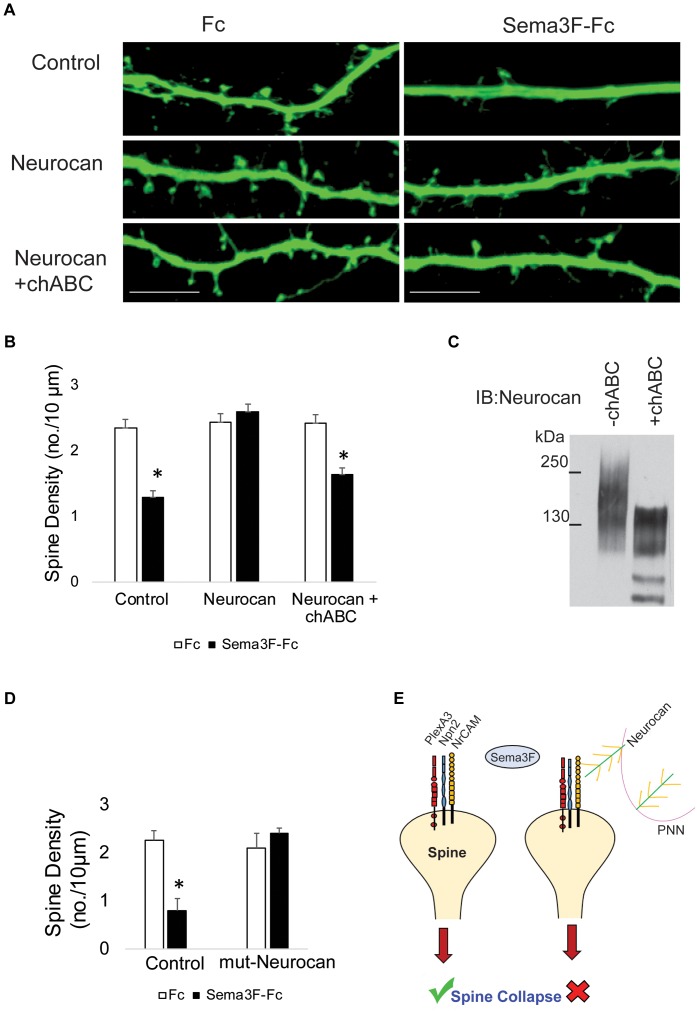
Enzymatic digestion of Neurocan GAG chains with chondroitinase ABC decreases its ability to inhibit Sema3F-induced spine retraction. **(A)** Images showing spines on apical dendrites from cortical neurons (EGFP, green) in culture treated with Fc or Sema3F-Fc. Neurocan blocked Sema3F-mediated spine retraction, whereas chABC-treated Neurocan was not effective. Scale bar = 10 μm. **(B)** Quantification of experiment in panel **A** shows a significant reduction in mean spine density of control neurons treated with Sema3F-Fc compared to Fc. Sema3F-induced spine retraction was fully blocked by 20 nM Neurocan, as well as by chABC-digested Neurocan (^∗^*p* < 0.05, *t*-test; *n* = 3, 10 neurons per condition). **(C)** Immunoblotting of Neurocan before and after treatment with chABC to remove GAG chains. A shift in apparent molecular size of chABC-treated Neurocan was observed, reflecting a decrease in GAG content. **(D)** Mouse cortical neurons with and without pre-treatment with recombinant mutNeurocan lacking the C-terminal sushi domain (20 nM, 30 min) showed the mouse Neurocan fragment inhibited Sema3F-Fc induced spine retraction. **(E)** Model showing that interaction of the PNN protein Neurocan with NrCAM on the surface of dendritic spines in cortical pyramidal neurons terminates Sema3F-induced dendritic spine remodeling during postnatal maturation. Neurocan core protein is depicted in green with yellow GAG chains. The Sema3F receptor complex is composed of NrCAM (yellow), Npn2 (blue), and PlexA3 (red) subunits.

CSPGs have been shown to restrict plasticity of cortical and hippocampal neurons, and digestion of their associated GAG chains with chondroitinase ABC (chABC) increases spine dynamics and density (Pizzorusso et al., 2002; Orlando et al., 2012; de Vivo et al., 2013). Therefore, we postulated that Neurocan GAG chains may be critical for inhibiting Sema3F-induced spine retraction. To test this hypothesis, GAG chains present on recombinant Neurocan were digested with chABC, as described (Sullivan et al., 2018). Chondroitinase-digested Neurocan was compared with untreated Neurocan for inhibition of Sema3F-Fc induced spine retraction in cortical neuron cultures. Results showed that chABC-treated Neurocan (20 nM, 30 min) did not block Sema3F-induced spine retraction, whereas untreated Neurocan effectively inhibited spine retraction compared to the untreated control (**Figures 5A,B**). GAG removal from Neurocan was confirmed on Western blots by a shift in GAG-modified Neurocan to lower molecular weight and band narrowing (**Figure 5C**).

Neurocan binds to the related adhesion molecule L1 through the C-terminal sushi domain, which contains about half of the GAG chains present in Neurocan (Oleszewski et al., 2000). To assess the role of the Neurocan sushi domain in NrCAM-mediated spine retraction, a recombinant mouse Neurocan fragment lacking the sushi domain (mutNeurocan) was assayed for inhibition of Sema3F-induced spine retraction in cortical neuron cultures. Neurocan lacking the sushi domain effectively inhibited Sema3F-mediated spine retraction, indicating that NrCAM, unlike L1, does not require the sushi domain or GAG-modification of the sushi region (**Figure 5D**).

## Discussion

We report here that Neurocan, one of the first CSPGs to be expressed in the maturing neocortex (Carulli et al., 2010), is expressed coordinately with Sema3F in postnatal and adult mouse brain in close apposition to dendritic spines and axon terminals. Neuronal spine retraction and cell binding studies showed that Neurocan interacts with NrCAM and inhibits Sema3F-mediated dendritic spine elimination, providing insight into the molecular basis of PNN-mediated restriction of synaptic plasticity. Our results support a model (**Figure 5E**) in which NrCAM/Npn2/PlexA3 functions as a holoreceptor complex for Sema3F that prunes excess dendritic spines from pyramidal neuron subpopulations during postnatal stages of active spine remodeling in the cerebral cortex. As PNNs arise during subsequent maturation, Neurocan within the PNN meshwork interacts with NrCAM on dendritic spines to terminate Sema3F-mediated spine pruning.

In postnatally developing areas of the mouse neocortex (MFC and V1), Neurocan was localized around processes, spines, and soma of Nex1-expressing postmitotic pyramidal neurons, then declined to lower levels in adulthood. As shown by immuno-electron microscopy of the frontal cortex (P18), Neurocan was prominent at plasma membranes and extracellular space, often in proximity to spines, axon terminals, and postsynaptic densities. Previously, we showed that Neurocan was also localized to perisomatic axon terminals and soma of inhibitory interneurons in the MFC (Sullivan et al., 2018). The present findings are in agreement with PNN localization at the surface of pyramidal cells in adult rat V1 and MFC (Alpar et al., 2006) and near dendritic spines in mouse hippocampus (Carstens et al., 2016). In a functional assay for Sema3F signaling in cortical neuron cultures, we found that Neurocan inhibited spine retraction on apical dendrites of pyramidal neurons. Similarly, in a model cell assay for Sema3F-induced repulsion, Neurocan inhibited morphological retraction through Sema3F receptor subunits NrCAM, Npn2, and PlexA3. Our culture experiments are in accord with two-photon live imaging studies in adult mouse visual cortex, which demonstrated that CSPGs inhibited spine dynamics and constrained spine morphology, and these effects were reversed by GAG chain degradation (Pizzorusso et al., 2002; de Vivo et al., 2013). The present findings suggest that Neurocan, possibly in conjunction with other CSPGs may contribute to terminating the highly active spine remodeling of juvenile cortical pyramidal neurons during the transition to adulthood.

Cell binding and ELISA assays showed that Neurocan was able to bind NrCAM within its extracellular region, which engages in homophilic and heterophilic interactions. chABC perturbed the ability of Neurocan to block Sema3F-mediated spine pruning in cultured neurons, indicating that GAG chains mediated this inhibition. Our binding results with recombinant proteins support radioimmunoassay studies showing binding of full length NrCAM purified from mouse brain to Neurocan (Grumet and Sakurai, 1996; Milev et al., 1998). Our experiments with the NrCAM extracellular domain protein fused to Fc further demonstrate that Neurocan interacts with the extracellular region of NrCAM sufficiently to impair neuronal function.

A limitation of the present work is that it is not known how NrCAM association with Neurocan inhibits spine remodeling, such as by altering upstream interactions or downstream signaling. Our results clearly showed that Neurocan did not interact with the upstream ligand Sema3F. Unlike Sema3A, which associates with the CSPGs versican and aggrecan in the brain extracellular matrix (Vo et al., 2013), Sema3F-Fc did not bind purified Neurocan, thus upstream sequestration of Sema3F is unlikely be responsible for inhibiting spine remodeling. We recently reported that NrCAM, Npn2, and PlexA3 form a complex for Sema3F required for spine retraction (Mohan et al., 2018). Neurocan binding might alter the conformation of NrCAM within the holoreceptor complex to prevent Sema3F-induced changes in receptor function. Such an alteration could be subtle, because Neurocan did not perturb the ability of NrCAM to co-immunoprecipitate with Npn2 from transfected HEK293 cells (not shown). The carboxy-terminal sushi domain of Neurocan, which mediates binding to L1-CAM (Oleszewski et al., 2000), was dispensable for inhibiting NrCAM-dependent spine retraction, suggesting a different mode of binding to NrCAM. Currently, little is known about Sema3F-induced signaling through PlexA3 in dendritic spines, future studies will be needed to address the possibility that Neurocan influences downstream pathways.

Neurocan and other CSPGs in PNNs have been reported to associate with Ig-class adhesion molecules, including NCAM, L1, NgCAM, Neurofascin, and TAG1, as well as integrins (Milev et al., 1998; Oleszewski et al., 2000; Falk et al., 2004; Hedstrom et al., 2007). Thus, Neurocan could have multiple targets in developing neurons, some of which may contribute to spine remodeling. For example, an inhibitory role for Neurocan was recently identified in terminating postnatal remodeling of GABAergic axon terminals in prefrontal cortex to stabilize perisomatic inhibitory synapses (Sullivan et al., 2018). Neurocan binds the NCAM Ig2 domain and competitively inhibits NCAM-EphA3 association to prevent ephrinA5/EphA3 signaling and axonal repulsion.

An inhibitory function of Neurocan in Sema3F-mediated spine pruning in the adolescent brain, suggested by our *in vitro* studies, might serve to protect a subpopulation of cortical pyramidal neurons from over-pruning of dendritic spines. Other mechanisms of synaptic elimination involve astrocyte-mediated phagocytosis through engulfment receptors (MerTK and MEGF10), microglia-mediated pruning (C1q, C3, and CX3CR1) (Chung et al., 2015) and autophagy through mTOR signaling (Huber et al., 2015). The involvement of Neurocan in regulating synapse remodeling on both excitatory and inhibitory neurons makes it an important candidate molecule for neurodevelopmental disorders with aberrant spine and synapse numbers that could impact cortical excitatory/inhibitory balance. In schizophrenia, PNNs are notably altered in human prefrontal cortex (Berretta et al., 2015) where dendritic spine density is markedly diminished (Garey et al., 1998; Glausier and Lewis, 2013; Moyer et al., 2015; MacDonald et al., 2017), and Neurocan is a candidate locus for schizophrenia, bipolar, and other neurological disorders (Cichon et al., 2011; Muhleisen et al., 2012; Schultz et al., 2014; Wang et al., 2016).

In conclusion, we provide evidence that the PNN protein Neurocan acts as a developmental brake for spine remodeling mediated by Sema3F and NrCAM in the maturing mouse neocortex.

## Author Contributions

VM conducted most of the experiments, analyzed the results, and contributed to writing the manuscript. EW performed the pull-down experiments and assisted in immunostaining. IG and SD performed the ELISA. RW and KP performed the immuno-EM experiments. BD assisted in neuronal cultures. AT assisted with the Neurocan binding experiments. PM designed the study, supervised the research, analyzed the EM data, and contributed to writing the manuscript.

## Conflict of Interest Statement

The authors declare that the research was conducted in the absence of any commercial or financial relationships that could be construed as a potential conflict of interest.

## References

[B1] Agarwal A. Dibaj P. Kassmann C. M. Goebbels S. Nave K. A. Schwab M. H. (2012). In vivo imaging and noninvasive ablation of pyramidal neurons in adult NEX-CreERT2 mice. *Cereb. Cortex* 22 1473–1486. 10.1093/cercor/bhr214 21880656

[B2] Alpar A. Gartner U. Hartig W. Bruckner G. (2006). Distribution of pyramidal cells associated with perineuronal nets in the neocortex of rat. *Brain Res.* 1120 13–22. 10.1016/j.brainres.2006.08.069 16996045

[B3] Berretta S. Pantazopoulos H. Markota M. Brown C. Batzianouli E. T. (2015). Losing the sugar coating: potential impact of perineuronal net abnormalities on interneurons in schizophrenia. *Schizophr. Res.* 167 18–27. 10.1016/j.schres.2014.12.040 25601362 PMC4504843

[B4] Bhattacharyya S. Zhang X. Feferman L. Johnson D. Tortella F. C. Guizzetti M. (2015). Decline in arylsulfatase B and Increase in chondroitin 4-sulfotransferase combine to increase chondroitin 4-sulfate in traumatic brain injury. *J. Neurochem.* 134 728–739. 10.1111/jnc.13156 25943740 PMC4516630

[B5] Bian W. J. Miao W. Y. He S. J. Qiu Z. Yu X. (2015). Coordinated spine pruning and maturation mediated by inter-spine competition for Cadherin/Catenin complexes. *Cell* 162 808–822. 10.1016/j.cell.2015.07.018 26255771

[B6] Carstens K. E. Phillips M. L. Pozzo-Miller L. Weinberg R. J. Dudek S. M. (2016). perineuronal nets suppress plasticity of excitatory synapses on CA2 pyramidal neurons. *J. Neurosci.* 36 6312–6320. 10.1523/JNEUROSCI.0245-16.2016 27277807 PMC4899529

[B7] Carulli D. Foscarin S. Faralli A. Pajaj E. Rossi F. (2013). Modulation of semaphorin3A in perineuronal nets during structural plasticity in the adult cerebellum. *Mol. Cell. Neurosci.* 57 10–22. 10.1016/j.mcn.2013.08.003 23999154

[B8] Carulli D. Pizzorusso T. Kwok J. C. Putignano E. Poli A. Forostyak S. (2010). Animals lacking link protein have attenuated perineuronal nets and persistent plasticity. *Brain* 133 2331–2347. 10.1093/brain/awq145 20566484

[B9] Chung W. S. Welsh C. A. Barres B. A. Stevens B. (2015). Do glia drive synaptic and cognitive impairment in disease? *Nat. Neurosci.* 18 1539–1545. 10.1038/nn.4142 26505565 PMC4739631

[B10] Cichon S. Muhleisen T. W. Degenhardt F. A. Mattheisen M. Miro X. Strohmaier J. (2011). Genome-wide association study identifies genetic variation in neurocan as a susceptibility factor for bipolar disorder. *Am. J. Hum. Genet.* 88 372–381. 10.1016/j.ajhg.2011.01.017 21353194 PMC3059436

[B11] de Vivo L. Landi S. Panniello M. Baroncelli L. Chierzi S. Mariotti L. (2013). Extracellular matrix inhibits structural and functional plasticity of dendritic spines in the adult visual cortex. *Nat. Commun.* 4:1484. 10.1038/ncomms2491 23403561

[B12] Demyanenko G. P. Mohan V. Zhang X. Brennaman L. H. Dharbal K. E. Tran T. S. (2014). Neural cell adhesion molecule NrCAM regulates semaphorin 3F-Induced dendritic spine remodeling. *J. Neurosci.* 34 11274–11287. 10.1523/JNEUROSCI.1774-14.2014 25143608 PMC4138338

[B13] Dick G. Tan C. L. Alves J. N. Ehlert E. M. Miller G. M. Hsieh-Wilson L. C. (2013). Semaphorin 3A binds to the perineuronal nets via chondroitin sulfate type E motifs in rodent brains. *J. Biol. Chem.* 288 27384–27395. 10.1074/jbc.M111.310029 23940048 PMC3779733

[B14] Einarsdottir E. Peyrard-Janvid M. Darki F. Tuulari J. J. Merisaari H. Karlsson L. (2017). Identification of NCAN as a candidate gene for developmental dyslexia. *Sci. Rep.* 7:9294. 10.1038/s41598-017-10175-7 28839234 PMC5570950

[B15] Falk J. Thoumine O. Dequidt C. Choquet D. Faivre-Sarrailh C. (2004). NrCAM coupling to the cytoskeleton depends on multiple protein domains and partitioning into lipid rafts. *Mol. Biol. Cell* 15 4695–4709. 10.1091/mbc.e04-03-0171 15254265 PMC519160

[B16] Flanagan J. G. Cheng H. J. (2000). Alkaline phosphatase fusion proteins for molecular characterization and cloning of receptors and their ligands. *Methods Enzymol.* 327 198–210. 10.1016/S0076-6879(00)27277-7 11044984

[B17] Friedlander D. R. Milev P. Karthikeyan L. Margolis R. K. Margolis R. U. Grumet M. (1994). The neuronal chondroitin sulfate proteoglycan Neurocan binds to the neural cell adhesion molecules Ng-CAM/L1/NILE and N-CAM, and inhibits neuronal adhesion and neurite outgrowth. *J. Cell Biol.* 125 669–680. 10.1083/jcb.125.3.669 7513709 PMC2119998

[B18] Garey L. J. Ong W. Y. Patel T. S. Kanani M. Davis A. Mortimer A. M. (1998). Reduced dendritic spine density on cerebral cortical pyramidal neurons in schizophrenia [see comments]. *J. Neurol. Neurosurg. Psychiatry* 65 446–453. 10.1136/jnnp.65.4.446 9771764 PMC2170311

[B19] Glausier J. R. Lewis D. A. (2013). Dendritic spine pathology in schizophrenia. *Neuroscience* 251 90–107. 10.1016/j.neuroscience.2012.04.044 22546337 PMC3413758

[B20] Gogolla N. Caroni P. Luthi A. Herry C. (2009). Perineuronal nets protect fear memories from erasure. *Science* 325 1258–1261. 10.1126/science.1174146 19729657

[B21] Grumet M. Sakurai T. (1996). Heterophilic interactions of the neural cell adhesion molecules Ng-CAM and Nr-CAM with neural receptors and extracellular matrix proteins. *Semin. Neurosci.* 8 379–389. 10.1006/smns.1996.0046

[B22] Hedstrom K. L. Xu X. Ogawa Y. Frischknecht R. Seidenbecher C. I. Shrager P. (2007). Neurofascin assembles a specialized extracellular matrix at the axon initial segment. *J. Cell Biol.* 178 875–886. 10.1083/jcb.200705119 17709431 PMC2064550

[B23] Hensch T. K. (2005). Critical period plasticity in local cortical circuits. *Nat. Rev. Neurosci.* 6 877–888. 10.1038/nrn1787 16261181

[B24] Huber K. M. Klann E. Costa-Mattioli M. Zukin R. S. (2015). Dysregulation of mammalian target of rapamycin signaling in mouse models of Autism. *J. Neurosci.* 35 13836–13842. 10.1523/JNEUROSCI.2656-15.2015 26468183 PMC4604222

[B25] Huttenlocher P. R. (1979). Synaptic density in human frontal cortex - developmental changes and effects of aging. *Brain Res.* 163 195–205. 10.1016/0006-8993(79)90349-4 427544

[B26] Hylin M. J. Orsi S. A. Moore A. N. Dash P. K. (2013). Disruption of the perineuronal net in the hippocampus or medial prefrontal cortex impairs fear conditioning. *Learn. Mem.* 20 267–273. 10.1101/lm.030197.112 23592037 PMC3630486

[B27] MacDonald M. L. Alhassan J. Newman J. T. Richard M. Gu H. Kelly R. M. (2017). Selective loss of smaller spines in Schizophrenia. *Am. J. Psychiatry* 174 586–594. 10.1176/appi.ajp.2017.16070814 28359200 PMC5800878

[B28] McAllister A. K. (2007). Dynamic aspects of CNS synapse formation. *Annu. Rev. Neurosci.* 30 425–450. 10.1146/annurev.neuro.29.051605.112830 17417940 PMC3251656

[B29] Milev P. Chiba A. Haring M. Rauvala H. Schachner M. Ranscht B. (1998). High affinity binding and overlapping localization of neurocan and phosphacan/protein-tyrosine phosphatase-zeta/beta with tenascin-R, amphoterin, and the heparin-binding growth-associated molecule. *J. Biol. Chem.* 273 6998–7005. 10.1074/jbc.273.12.6998 9507007

[B30] Miyata S. Kitagawa H. (2017). Formation and remodeling of the brain extracellular matrix in neural plasticity: roles of chondroitin sulfate and hyaluronan. *Biochim. Biophys. Acta* 1861 2420–2434. 10.1016/j.bbagen.2017.06.010 28625420

[B31] Mohan V. Sullivan C. S. Guo J. Wade S. D. Majumder S. Agarwal A. (2018). Temporal regulation of dendritic spines through NrCAM-semaphorin3f receptor signaling in developing cortical pyramidal neurons. *Cereb. Cortex* 10.1093/cercor/bhy004 [Epub ahead of print]. 29415226 PMC6499012

[B32] Moyer C. E. Shelton M. A. Sweet R. A. (2015). Dendritic spine alterations in schizophrenia. *Neurosci. Lett.* 601 46–53. 10.1016/j.neulet.2014.11.042 25478958 PMC4454616

[B33] Muhleisen T. W. Mattheisen M. Strohmaier J. Degenhardt F. Priebe L. Schultz C. C. (2012). Association between schizophrenia and common variation in neurocan (NCAN), a genetic risk factor for bipolar disorder. *Schizophr. Res.* 138 69–73. 10.1016/j.schres.2012.03.007 22497794

[B34] Oleszewski M. Gutwein P. Von Der Lieth W. Rauch U. Altevogt P. (2000). Characterization of the L1-neurocan-binding site. Implications for L1-L1 homophilic binding. *J. Biol. Chem.* 275 34478–34485. 10.1074/jbc.M004147200 10934197

[B35] Orlando C. Ster J. Gerber U. Fawcett J. W. Raineteau O. (2012). Perisynaptic chondroitin sulfate proteoglycans restrict structural plasticity in an integrin-dependent manner. *J. Neurosci.* 32 18009–18017, 18017a. 10.1523/JNEUROSCI.2406-12.2012 23238717 PMC6621736

[B36] Peng S. S. Tran T. S. (2017). Regulation of cortical dendrite morphology and spine organization by secreted semaphorins: a primary culture approach. *Methods Mol. Biol.* 1493 209–222. 10.1007/978-1-4939-6448-2_15 27787853

[B37] Petanjek Z. Judas M. Simic G. Rasin M. R. Uylings H. B. Rakic P. (2011). Extraordinary neoteny of synaptic spines in the human prefrontal cortex. *Proc. Natl. Acad. Sci. U.S.A.* 108 13281–13286. 10.1073/pnas.1105108108 21788513 PMC3156171

[B38] Pizzorusso T. Medini P. Berardi N. Chierzi S. Fawcett J. W. Maffei L. (2002). Reactivation of ocular dominance plasticity in the adult visual cortex. *Science* 298 1248–1251. 10.1126/science.1072699 12424383

[B39] Pyka M. Wetzel C. Aguado A. Geissler M. Hatt H. Faissner A. (2011). Chondroitin sulfate proteoglycans regulate astrocyte-dependent synaptogenesis and modulate synaptic activity in primary embryonic hippocampal neurons. *Eur. J. Neurosci.* 33 2187–2202. 10.1111/j.1460-9568.2011.07690.x 21615557

[B40] Rauch U. Karthikeyan L. Maurel P. Margolis R. U. Margolis R. K. (1992). Cloning and primary structure of neurocan, a developmentally regulated, aggregating chondroitin sulfate proteoglycan of brain. *J. Biol. Chem.* 267 19536–19547. 1326557

[B41] Schultz C. C. Muhleisen T. W. Nenadic I. Koch K. Wagner G. Schachtzabel C. (2014). Common variation in NCAN, a risk factor for bipolar disorder and schizophrenia, influences local cortical folding in schizophrenia. *Psychol. Med.* 44 811–820. 10.1017/S0033291713001414 23795679

[B42] Shen Y. Tenney A. P. Busch S. A. Horn K. P. Cuascut F. X. Liu K. (2009). PTPsigma is a receptor for chondroitin sulfate proteoglycan, an inhibitor of neural regeneration. *Science* 326 592–596. 10.1126/science.1178310 19833921 PMC2811318

[B43] Sullivan C. S. Gotthard I. Wyatt E. V. Bongu S. Mohan V. Weinberg R. J. (2018). Perineuronal net protein neurocan inhibits NCAM/EphA3 repellent signaling in GABAergic interneurons. *Sci. Rep.* 8:6143. 10.1038/s41598-018-24272-8 29670169 PMC5906663

[B44] Tran T. S. Rubio M. E. Clem R. L. Johnson D. Case L. Tessier-Lavigne M. (2009). Secreted semaphorins control spine distribution and morphogenesis in the postnatal CNS. *Nature* 462 1065–1069. 10.1038/nature08628 20010807 PMC2842559

[B45] Turner L. J. Hall A. (2006). Plexin-induced collapse assay in COS cells. *Methods Enzymol.* 406 665–676. 10.1016/S0076-6879(06)06052-6 16472696

[B46] Vo T. Carulli D. Ehlert E. M. Kwok J. C. Dick G. Mecollari V. (2013). The chemorepulsive axon guidance protein semaphorin3A is a constituent of perineuronal nets in the adult rodent brain. *Mol. Cell. Neurosci.* 56 186–200. 10.1016/j.mcn.2013.04.009 23665579

[B47] Wang P. Cai J. Ni J. Zhang J. Tang W. Zhang C. (2016). The NCAN gene: schizophrenia susceptibility and cognitive dysfunction. *Neuropsychiatr. Dis. Treat.* 12 2875–2883. 10.2147/NDT.S118160 27853371 PMC5104293

